# The Electronic Psychiatric Semi-structured Interview for Children and Adolescents (EPSI-C): development and psychometric evaluation of an open-access DSM-5-based diagnostic instrument

**DOI:** 10.1186/s13034-026-01094-5

**Published:** 2026-05-06

**Authors:** Susanne Olofsdotter, Melpomeni Dragou, Johan Isaksson, Kent W. Nilsson, Sofia Vadlin, Maria Hedqvist

**Affiliations:** 1 Center for Clinical Research Västmanland, Uppsala University, Västerås, S-721 89 Sweden; 2 Child and Adolescent Psychiatry Unit, Department of Medical Sciences, Uppsala University, Uppsala, Sweden; 3 Center of Neurodevelopmental Disorders (KIND), Centre for Psychiatry Research, Department of Women’s and Children’s Health, Karolinska Institutet & Stockholm Health Care Services, Region Stockholm, Stockholm, Sweden; 4 School of Health, Care and Social Welfare, Division of Public Health Sciences, Mälardalen University, Västerås, Sweden; 5 Department of Psychology, Uppsala University, Uppsala, Sweden

**Keywords:** EPSI-C, Diagnostic interview, Child and adolescent psychiatry, DSM-5, Content validity, Internal consistency, Digital implementation, eHealth

## Abstract

**Background:**

Standardized diagnostic interviews are essential tools for accurate assessment in child and adolescent psychiatry (CAP). However, many existing interviews are restricted by licensing costs, platform dependencies, limited clinical adaptability, or legal ambiguity regarding digital implementation. To address these limitations, the Electronic Psychiatric Semi-structured Interview for Children and Adolescents (EPSI-C) was developed as an open-access, platform-independent instrument aligned with the DSM-5. The modular structure, comprising 22 modules covering the full range of common psychiatric disorders, allows for both systematic and targeted administration. This instrument development and psychometric validation study evaluated the content validity and internal consistency of the EPSI-C.

**Methods:**

Content validity was assessed by ten independent experts who rated the relevance of the instrument for assessing DSM-5 diagnostic criteria. Additionally, experts rated six global aspects of clinical utility. Internal consistency was examined in a large naturalistic sample of 3,506 patients (mean age = 12.0 years, SD = 3.2, range 5–18 years; 54.5% girls) assessed with a digital implementation of the EPSI-C during routine outpatient CAP assessments in Sweden.

**Results:**

The EPSI-C demonstrated excellent scale-level average content validity for both the screening sections (S-CVI/Ave = 0.95) and full modules (S-CVI/Ave = 0.94). Expert consensus was high (90–100%) regarding overall clinical utility. Internal consistency was acceptable to excellent (α range 0.71 – 0.90) for 87.5% of eligible modules. Lower consistency was observed for modules assessing psychotic disorders (α = 0.53) and anorexia nervosa (α = 0.49), reflecting limited DSM-5 criteria and sub-threshold presentations.

**Conclusions:**

The findings support the foundational psychometric properties of the EPSI-C and indicate its clinical feasibility within a high-volume, naturalistic CAP outpatient setting. The current study establishes content validity and internal consistency, while criterion validity remains to be evaluated. As an open-access, modular instrument, EPSI-C offers a scalable solution for the digital implementation of standardized diagnostic interviews in routine clinical practice.

**Supplementary Information:**

The online version contains supplementary material available at 10.1186/s13034-026-01094-5.

## Background

Accurate psychiatric diagnosis in children and adolescents is fundamental to evidence-based treatment and service planning. However, diagnostic assessment in child and adolescent psychiatry (CAP) is notably variable, often relying on unstructured clinical interviews with poor reliability and low agreement between clinicians [1–4]. Insufficient diagnostic precision and overlooked comorbidity can lead to inappropriate treatment selection, prolonged wait times, and inefficient care pathways [5, 6].

Broad, standardized interviews are widely recognized as essential tools for improving diagnostic accuracy. By covering symptoms and diagnostic criteria comprehensively, these tools facilitate the detection of comorbidities and provide a robust foundation for treatment planning [1, 4, 6–8]. Prominent instruments, such as the Schedule for Affective Disorders and Schizophrenia for School-Age Children (K-SADS) [9], have demonstrated strong psychometric properties and serve as the “gold standard” in research settings.

Despite the availability of established interviews, a persistent implementation gap remains in routine practice. Many standardized interviews were originally developed for research contexts and are often constrained by general barriers such as licensing costs or time-consuming, rigid formats [4]. While digital administration may offer efficiency [1, 4], developers often restrict electronic use to proprietary, closed software environments. These software platforms are frequently governed by pay-per-use licensing or subscription models, which introduce recurring costs that scale with patient volume. Furthermore, such systems often lack the interoperability required for independent data export or integration into local electronic health records (EHR), creating data silos. Beyond technical constraints, navigating the legal requirements for digital adaptation presents a significant logistical hurdle. For instruments that are free to use in paper format, information on usage rights for digital adaptation may be insufficient or hard to find, making it difficult for CAP services to ensure legal compliance [10]. Consequently, services are often forced to choose between fragmented, high-cost platforms for electronic administration or manual paper-based workflows. Together, these technical, financial, and legal barriers hinder widespread implementation and equitable access to evidence-based assessment in public mental health services [4, 10].

To address these limitations, the Electronic Psychiatric Semi-structured Interview for Children and Adolescents (EPSI-C) was developed to provide an open-access, platform-independent diagnostic instrument that directly operationalizes diagnostic criteria according to the fifth edition of the Diagnostic and Statistical Manual of Mental Disorders (DSM-5) [11, 12]. It consists of a suicidality risk module and 21 diagnostic modules, covering the full range of common child and adolescent psychiatric disorders, including emotional, neurodevelopmental, eating, and disruptive behavior disorders. The EPSI-C’s modular structure allows for both systematic and targeted administration where diagnostic modules are selected based on clinical indication informed by prior assessments, e.g., intake questionnaires [13]. This flexibility addresses the practical demands of routine clinical practice, offering an alternative to rigid, time-consuming formats. To eliminate legal and financial ambiguity, the EPSI-C includes explicit, pre-authorized permission for implementation into both digital and paper-based local systems without licensing fees or the requirement of specific software. By providing these clear conditions from the outset, the EPSI-C removes the need for clinical services to engage in time-consuming legal inquiries or negotiate usage terms with developers. Since 2021, the EPSI-C has been integrated into routine CAP practice within two independent healthcare regions in Sweden.

Before a new instrument is disseminated for wider use, it is essential to formally establish its psychometric properties. Two foundational aspects of such evaluations are content validity and internal consistency. Content validity reflects the extent to which the interview content represents the diagnostic domains it is intended to assess and is typically evaluated through expert judgment of relevance and representativeness [14, 15]. Internal consistency reflects the degree to which the individual elements of an instrument function together as coherent indicators of the same underlying construct [16, 17]. If the instrument assesses multiple constructs, as in the case of broad, semi-structured interviews such as the EPSI-C, internal consistency should be calculated separately for each construct [16]. Importantly, psychometric properties are not inherent attributes of an instrument but are contingent upon the specific population and context in which the instrument is applied [18]. Evaluating these properties within the heterogeneous and diagnostically complex population of routine clinical CAP care is therefore essential for ensuring ecological validity. This study aims to describe the development of the EPSI-C and evaluate its foundational psychometric properties, specifically: (i) content validity through expert relevance ratings, and (ii) internal consistency within a large naturalistic cohort of children and adolescents, assessed within CAP under routine clinical conditions.

## Methods

### Development and structure of the EPSI-C

The EPSI-C was developed between 2019 and 2021 by a multidisciplinary working group of clinician-scientists in a collaboration between the CAP clinic and the Center for Clinical Research in Västmanland County, Sweden. The EPSI-C was designed as a clinician-administered psychiatric diagnostic interview for children and adolescents aged 6–17 years. Its primary objective was to provide an open-access, platform-independent diagnostic tool that directly operationalizes the DSM-5 diagnostic criteria into standardized interview items. The platform-independent design was specifically intended to enable seamless implementation into local digital systems. Development followed an iterative process that included defining the diagnostic scope, drafting 21 disorder-specific modules (see Table 1 for a complete list), each consisting of a screening section and a full module, and a comprehensive suicidality risk module, and incorporating feedback from experienced CAP clinicians. Development was informed by established models of structured diagnostic interviewing while aiming to maximize clinical usability. Pilot testing was conducted in routine CAP settings to refine clarity and feasibility. Following pilot testing, the EPSI-C was introduced into routine assessment procedures at the CAP clinic in Västmanland County. During the initial implementation phase, a quality check of interrater reliability was conducted on 15 recorded interviews. This preliminary assessment focused on binary diagnostic outcomes for each module and indicated high overall agreement (99%), largely reflecting consensus on the absence of diagnosis.

Administering the EPSI-C requires clinical experience in diagnostic assessment and knowledge of the developmental presentations of mental disorders. Interviews may be conducted jointly with the child and parents or separately at the discretion of the interviewer. A thorough anamnestic interview is recommended prior to administration. Based on clinical use within CAP services in Sweden, administration time typically ranges from 30 to 90 min, depending on case complexity and the number of full modules completed. Each disorder-specific module begins with brief guidance for the interviewer, followed by a screening section identifying probable cases. If the screen is positive, the full module is administered to evaluate each relevant DSM-5 criterion via suggested, yet flexible, probe questions. The interviewer judges whether the criterion is met, partially met, or not met, based on a synthesis of information obtained during the interview. While the EPSI-C provides a standardized framework for information structuring, the final decision on diagnostic status relies on clinical judgment and integration, where interview findings are synthesized with anamnestic data and other relevant clinical information. This structure supports systematic assessment while preserving the necessary clinical flexibility.

The EPSI-C’s open-access and platform-independent design facilitates integration into diverse local digital infrastructures. CAP services may implement the tool in a paper-based format or digitize the content into their preferred digital platform. Once digitized, the fixed alignment with the DSM-5 facilitates the automated summary of results and potential diagnoses by programming the system to apply DSM-5 diagnostic algorithms. The original Swedish version of the EPSI-C, along with an English translation and a result summary form mapping EPSI-C items to DSM-5 criteria, are accessible through the Uppsala University website of the Center for Clinical Research Västmanland [19]. A sample module (Generalized Anxiety Disorder) is provided in English in Supplementary File 1.

### Participants and procedures

#### Expert panel and content validity procedure

The expert panel consisted of ten clinicians (six psychologists, three child and adolescent psychiatrists, and one psychiatric nurse specialist) recruited from two separate CAP clinics in Sweden. The experts had a mean of 9.6 years in CAP clinical practice (range 2–20 years) and substantial experience with standardized diagnostic interviews such as the MINI-KID [20] and the K-SADS [9], and were well-acquainted with the EPSI-C through its use in their clinical work. All experts were independent of the research team and had no role in the instrument’s conceptualization, item formulation, or authorship. No formal research training or calibration was provided. Evaluations were performed independently based on individual professional judgment. The procedure evaluated the content validity of the EPSI-C relative to DSM-5 diagnostic constructs. Using a structured electronic form, experts independently rated content relevance on a four-point scale (1 = very low, 2 = low, 3 = high, 4 = very high). For each module, experts provided two ratings based on their overall impression of its distinct structural components. First, the screening section was rated for its relevance in identifying core symptoms and determining the need for further assessment. Second, the full module was rated for overall relevance of all included items and alignment with DSM-5 diagnostic criteria. For the suicidality module, the experts provided a single rating of the overall relevance of the content for identifying suicidality and self-harm. This approach allowed experts to evaluate the modules as cohesive units, including the probing questions, rather than requiring item-by-item assessment. Finally, experts provided global evaluations of the full interview regarding overall relevance, clarity, coverage, structure, usability, and clinical credibility, using the same four-point scale.

#### Clinical sample and internal consistency procedure

Data for assessing internal consistency were retrieved from a repository at an outpatient intake unit within the CAP clinic in Västmanland County, Sweden. The unit manages all new referrals to the clinic, most of which concern suspected neurodevelopmental disorders. Under the county’s stepped-care model, youth with mild mental health concerns or primary conduct and substance use disorders are directed to primary care or social services, respectively. Patients with specific or acute conditions (e.g., eating disorders, bipolar disorder, psychotic disorders) bypass the intake unit and are routed directly to specialized units. This organization of care is reflected in the intake unit’s standardized assessment protocol. All patients undergo systematic screening with a core set of EPSI-C modules, including those for anxiety disorders, depression, posttraumatic stress disorder, obsessive-compulsive disorder, psychosis, ADHD, autism, and suicidality. Other modules are screened only when clinically indicated. Consistent with the EPSI-C structure, full diagnostic modules are administered conditionally, triggered only upon a positive screening result. The dataset comprised 3,506 unique, consecutive patients (mean age = 12.0 years, SD = 3.2, range 5–18 years; 54.5% girls). Each patient was assessed with the EPSI-C on a single occasion as part of routine procedures during face-to-face visits between November 2021 and December 2024. Assessments were conducted as joint interviews involving the patient, their parents, and a clinician, with portions of the interview conducted separately with the patient when clinically appropriate. There were no formal exclusion criteria; the joint format and use of language interpreters accommodated patients with cognitive, verbal, or linguistic barriers. Clinicians used the clinic’s existing digital assessment platform during administration of the EPSI-C to record their ratings of criteria fulfillment.

### Statistical analysis

Statistical analyses were performed using IBM SPSS Statistics (version 28). Content validity was assessed by calculating the item-level content validity index (I-CVI) in accordance with the method described by Polit, Beck, and Owen [14]. Experts provided overall relevance ratings for the structural components of each module, i.e., the screening section and the full module, rather than for individual items; these components served as the units of analysis for CVI calculations. The I-CVI was calculated as the proportion of experts assigning a relevance rating of 3 (high) or 4 (very high) to each component [14]. The overall content validity (scale-level content validity; S-CVI/Ave) was calculated by averaging the I-CVI values across all modules [21]. Following recommendations for a panel of 10 experts, I-CVI values of 0.70 were considered acceptable, whereas S-CVI/Ave values of ≥ 0.90 and I-CVI values of ≥ 0.78 indicated excellent content validity, mathematically ensuring excellent chance-adjusted agreement (modified kappa) greater than 0.75 [14, 22].

For the global evaluations of the full EPSI-C (overall relevance, clarity, coverage, structure, usability, and clinical credibility), the percentage of experts assigning a rating of 3 (high) or 4 (very high) was calculated for each aspect.

Internal consistency for each EPSI-C module was estimated using Cronbach’s alpha (α). Analyses included all items within each module, encompassing the screening section, the subsequent full module with diagnostic criteria, and symptom-specific check-boxes used to record the presence of various clinical manifestations (e.g., specific physical symptoms in panic disorder or the variety of tics in Tourette’s disorder). Missing data were handled via listwise deletion. Following conventional thresholds, Cronbach’s α values ≥ 0.70, ≥ 0.80, and ≥ 0.90 were interpreted as acceptable, good, and excellent internal consistency, respectively [16, 23]. To ensure stable estimation, only modules with ≥ 30 complete cases were analyzed, leaving 16 of 22 modules eligible for analysis [24]. Ineligible modules represented conditions with low prevalence at the intake unit. The number of patients with positive screening results, excluded patients due to missing data, and final analysis sample for each module is detailed in Supplementary File 2.

## Results

### Content validity

All modules demonstrated excellent content validity (I-CVI ≥ 0.78) for both the screening sections and the full modules, with the exception of the anorexia nervosa module (Table 1). While lower, the anorexia nervosa module (I-CVI = 0.70) remained above the established threshold for acceptability. The scale-level average content validity for all modules (S-CVI/Ave) was 0.95 for the screening sections and 0.94 for the full modules, meeting the criterion for excellent overall content validity. Expert evaluations of the six global aspects demonstrated a high degree of consensus. For five aspects—relevance, coverage, structure, usability, and clinical credibility—100% of experts provided ratings of either 3 (‘High’) or 4 (‘Very high’). For the aspect of clarity, 90% of experts provided such ratings, with one expert providing a rating of 2 (‘Low’). The distribution of ratings for ‘High’ versus ‘Very high’ was as follows: relevance (0/100%), clarity (40%/50%), coverage (30%/70%), structure (70%/30%), usability (0/100%), and clinical credibility (10/90%).

**Table 1:** Modules included in the EPSI-C and content validity indices for screening sections, full modules, and the overall instrument

Module	Screening section (I-CVI)	Full Module (I-CVI)
Specific phobia	1.00	1.00
Separation anxiety disorder	1.00	1.00
Social anxiety disorder	1.00	0.89
Panic disorder	0.80	0.90
Agoraphobia	0.80	1.00
Generalized anxiety disorder	1.00	1.00
Obsessive-compulsive disorder	1.00	1.00
PTSD	0.80	0.80
Major depressive episode	1.00	0.78
Persistent depression	1.00	1.00
Manic/hypomanic episode	1.00	1.00
Screen for psychotic disorders	0.89	0.78
Anorexia nervosa	0.70	0.70
Bulimia nervosa	1.00	1.00
Binge-eating disorder	0.90	1.00
ADHD	1.00	1.00
Autism spectrum disorder	1.00	1.00
Tourette’s disorder/tics	1.00	1.00
Oppositional defiant disorder	1.00	0.90
Conduct disorder	1.00	0.90
Screen for substance use disorders	1.00	1.00
Suicidality^*^	—	1.00
**S-CVI/Ave**	0.95	0.94

Note. I-CVI = item-level content validity index; S-CVI/Ave = scale-level content validity index (average I-CVI); ADHD = attention-deficit/hyperactivity disorder; PTSD = posttraumatic stress disorder; ^*^ The module does not include a screening section

### Internal consistency

The internal consistency (Cronbach’s α) for each module, including the number of patients and items included in the analyses, is presented in Table 2. Among the 16 modules with a minimum of 30 patients with complete data, the majority (*n* = 14; 87.5%) demonstrated acceptable to excellent internal consistency (Cronbach’s α 0.71–0.90). Lower estimates were observed for modules assessing anorexia nervosa (α = 0.49) and psychotic disorders (α = 0.53). For the suicidality module, internal consistency was estimated separately for two time frames, confirming stable reliability both within the past month and earlier.

**Table 2:** Internal consistency (Cronbach’s α) for EPSI-C modules

EPSI-C Module (no. of items included)^a^	Cronbach’s α	*n*
Specific phobia (6 items)	0.90	64
Separation anxiety disorder (11 items)	0.85	430
Social anxiety disorder (9 items)	0.83	997
Panic disorder (17 items)	0.75	495
Agoraphobia (13 items)	0.71	147
Generalized anxiety disorder (10 items)	0.73	787
Obsessive-compulsive disorder (7 items)	0.83	198
PTSD (32 items)	0.88	94
Major depressive episode (12 items)	0.83	1033
Persistent depression (10 items)	— (excluded, *n* < 30)	13
Manic/hypomanic episode (13 items)	— (excluded, *n* < 30)	20
Screen for psychotic disorders (8 items)	0.53	61
Anorexia nervosa (3 items)	0.49	41
Bulimia nervosa (6 items)	— (excluded, *n* < 30)	6
Binge-eating disorder (11 items)	— (excluded, *n* < 30)	3
ADHD (22 items)	0.90	2352
Autism spectrum disorder (10 items)	0.83	1408
Tourette’s disorder/Tics (23 items)	0.76	56
Oppositional defiant disorder (10 items)	0.84	74
Conduct disorder (18 items)	— (excluded, *n* < 30)	16
Screen for substance use disorder (38 items)	— (excluded, *n* < 30)	4
Suicidality past month/earlier^*b*^ (7/7 items)	0.83/0.86	3354/3350

^***a***^ Items included screening sections, full modules, and symptom checkboxes^*b*^ Estimates were calculated separately for two time frames: symptoms occurring within the past month/symptoms occurring earlier than the past month; ADHD = attention-deficit/hyperactivity disorder; PTSD = posttraumatic stress disorder.

## Discussion

This study describes the development of the Electronic Psychiatric Semi-structured Interview for Children and Adolescents (EPSI-C) and provides initial evidence for its content validity and internal structure within a CAP context. Expert evaluations demonstrated high content relevance across modules, with excellent overall content validity indices. In addition, consistently high expert ratings for global aspects suggest that the EPSI-C covers relevant clinical domains, features clear language and structure, and demonstrates high usability and clinical credibility. While internal consistency varied, the overall results indicate that the EPSI-C is a psychometrically sound tool for standardized DSM-5-based assessment in clinical practice.

Evaluations of psychiatric diagnostic interviews often prioritize analyses of inter-rater reliability over internal consistency, limiting direct comparisons with established interviews. However, the results for the EPSI-C ADHD module (α = 0.90) are comparable to the excellent internal reliability reported for the K-SADS ADHD module (McDonald’s omega for screening: ω = 0.89; supplement ω = 0.95) [25]. While α coefficients were adequate (0.71–0.90) for 87.5% of eligible modules, the lower values observed for the anorexia nervosa (α = 0.49) and the screen for psychotic disorders (α = 0.53) modules should be interpreted in light of Cronbach’s α’s known sensitivity to limited number of items and score variance, and the assumption of homogeneity [16, 18, 23]. First, the DSM-5 specifies only three diagnostic criteria for anorexia nervosa, resulting in a limited item pool for this EPSI-C module which inherently reduces the value of α [18, 23]. Second, the clinic’s stepped-care model routed patients with overt and acute symptoms of eating, psychotic, and bipolar disorders directly to specialized units, leading to analysis samples for these modules consisting of primarily sub-threshold cases with a restricted symptom range. This lack of score variability further attenuates α estimates [18]. Notably, even in samples without such triage, the internal consistency of DSM-5 criteria for anorexia nervosa has been reported as inadequate (α = 0.61) [26]. This suggests that the low α observed for this module reflects the inherent DSM-5 diagnostic structure rather than limitations of the study sample or the EPSI-C. Finally, Cronbach’s α’s assumption of homogeneity of what is being assessed is often at odds with the heterogeneous nature of psychiatric constructs, which can cause further underestimation of reliability [16]. While the use of Cronbach’s α has been criticized in favor of alternative metrics such as McDonald’s ω, recent research suggests that both perform similarly across diverse conditions, supporting α’s continued utility as a robust metric [27]. Reporting α within a naturalistic CAP population reflects how the internal coherence of the EPSI-C modules manifests under real-world conditions, ensuring transparency and providing an ecologically valid perspective on the instrument’s reliability.

Beyond its psychometric properties, the EPSI-C addresses significant practical barriers to the implementation of standardized diagnostic interviews in routine child and adolescent psychiatry. Unlike many established interviews constrained by licensing fees, platform dependencies, or rigid formats, the EPSI-C offers an open-access, platform-independent, and clinically flexible alternative. By demonstrating foundational evidence of its content validity and reliability in a naturalistic outpatient CAP setting, this study establishes the necessary groundwork for subsequent effectiveness and implementation research. 

### Strengths and limitations

A key strength of this study is the integration of independent expert evaluations with clinical data from a large, naturalistic cohort. Unlike validation studies restricted to controlled research settings, this study used data from routine, stepped-care outpatient services, ensuring high ecological validity. By capturing a broad range of clinical presentations and comorbidities typically encountered in everyday CAP outpatient practice, the findings demonstrate the performance of the EPSI-C under real-world conditions. The content validity assessment benefited from an independent and clinically grounded expert panel unaffiliated with the development of the EPSI-C yet experienced in its routine application. This design ensures that the content validity ratings reflect practical clinical relevance rather than theoretical assumptions, while recruitment from two separate clinics minimizes site-specific bias. Furthermore, the consistently high ratings indicated excellent chance-adjusted agreement (modified kappa > 0.75), underscoring the strong consensus among the independent experts [14]. Finally, the digital administration of the EPSI-C ensured adherence to the instrument’s gating logic and high data completeness.

Several limitations should be noted. First, the current study lacked a formal criterion validity assessment against established diagnostic instruments. However, the EPSI-C is not intended to function as an autonomous diagnostic tool. Instead, its role is complementary to clinical expertise and judgement, serving as a standardized framework for systematic coverage of diagnostic criteria and information structuring. Within this framework, clinical integration remains the decisive factor, as diagnostic validity ultimately relies on the clinician’s expertise to synthesize interview findings with anamnestic data, observations, and other relevant clinical information. Second, internal consistency was assessed only within symptomatic subsamples of patients who endorsed the initial screening items of each module, as dictated by the interview’s skip-logic structure. Furthermore, the use of a routine clinical sample within a stepped-care framework introduced a systematic selection bias. Local clinical pathways, where certain conditions were routed directly to specialized units, created a filtering effect, resulting in insufficient sample sizes for analyses of six modules assessing mood, eating, substance use, and conduct disorders. Finally, while an initial interrater reliability check indicated high overall agreement across modules, these preliminary findings should be interpreted with caution. Given the small sample size (*n* = 15) and high prevalence of negative diagnostic outcomes, these results provide only an initial indication of agreement. Further studies with larger, more diagnostically diverse samples are required to fully establish the instrument’s inter-rater reliability across the full clinical spectrum.

### Clinical and research implications

The EPSI-C provides a structured yet flexible framework suitable for routine child and adolescent psychiatry. Its modular architecture facilitates adaptation to diverse clinical workflows; while this study was conducted in a setting utilizing a standardized protocol for module selection, the instrument is equally suited for targeted administration where modules are selected strictly on the basis of clinical indication (e.g., guided by referral data, previsit questionnaires, or clinical observation). Future implementation research should compare these strategies to evaluate feasibility, clinician burden, and diagnostic yield.

Digital administration improves efficiency through automated data capture and immediate generation of structured reports based on DSM-5 criteria. By reducing administrative burden and time required for manual reporting, digital implementation addresses a primary barrier to the routine adoption of standardized diagnostic interviews [4].

Priorities for future research include establishing criterion validity against established interviews. Further work is also needed to examine the effectiveness and acceptability of the EPSI-C in routine settings. Of particular interest is evaluating diagnostic stability by comparing standardized assessment procedures using the EPSI-C versus nonstandardized assessment. Specifically, such research should investigate whether the EPSI-C reduces diagnostic flux, i.e., the frequency of subsequent diagnostic adjustments. Reduced flux would suggest a more accurate detection of mental disorders and comorbidities, addressing a major deficiency in unstandardized assessment. Moreover, successful implementation requires clinical acceptability across all stakeholders. Given the gaps identified in a recent review [1], more research is needed to explore the acceptability and user experience among a broad spectrum of healthcare professionals, including nurses and allied health staff, as well as patients and their parents. Evaluating user experience is essential to determine if a standardized assessment procedure with the EPSI-C is sustainable in routine practice. Finally, cross-cultural validation is required to establish the instrument’s utility beyond the Swedish context. The EPSI-C has already been translated into several languages, with ongoing pilot studies in additional European mental health care services aimed at evaluating its feasibility, reliability, and acceptability across diverse clinical settings.

## Conclusions

The EPSI-C facilitates standardized assessment in child and adolescent psychiatry by providing an open-access diagnostic interview specifically developed for routine care and increased efficiency through digital implementation. By design, it eliminates the financial, technical, and legal barriers typically associated with digital adaptation of established diagnostic interviews, offering a scalable alternative without licensing fees or specific software requirements. Pending further validation, this study establishes the foundational psychometric properties of the EPSI-C and indicates its clinical feasibility in high-volume settings. By enabling integration into local digital workflows, the EPSI-C promotes a broader adoption of standardized diagnostic assessments through more time-efficient administration.

## Supplementary Information

Below is the link to the electronic supplementary material.

Supplementary Material 1

Supplementary Material 2

## Data Availability

The datasets generated and/or analyzed during the current study are not publicly available due to ethical restrictions but are available from the corresponding author on reasonable request. Both the original Swedish version of the EPSI-C and an English translation, alongside a support document for summarizing EPSI-C results according to DSM-5 diagnostic criteria, are available as open-access resources through the Center for Clinical Research at Uppsala University: [Salve English – Centre for Clinical Research Västmanland – Uppsala University](https://www.uu.se/en/centre/clinical-research-vastmanland/research/salve-english(19).

## Abbreviations

ADHD: Attention-Deficit/Hyperactivity Disorder
CAP: Child and Adolescent Psychiatry
DSM-5: Diagnostic and Statistical Manual of Mental Disorders
EHR: Electronic health records
EPSI-C: Electronic Psychiatric Semi-structured Interview for Children and Adolescents
I-CVI: Item-level Content Validity Index
K-SADS: Schedule for Affective Disorders and Schizophrenia for School-Age Children
MINI-KID: Mini International Neuropsychiatric Interview for Children and Adolescents
PTSD: Post-Traumatic Stress Disorder
S-CVI/Ave: Scale-level Content Validity Index, Average

## References

[1] Basheer S, Tan SF, David R, Majumder P, Sayal K. Review Article: The acceptability and effectiveness of standardised diagnostic assessment approaches in children and young people’s mental health services – an updated systematic review. Child Adolesc Ment Health. 2025;30(4):364–74. 10.1111/camh.70007 . PubMed PMID: 40574522; PubMed Central PMCID: PMC12573065. 40574522 10.1111/camh.70007 PMC12573065

[2] Cook JR, Hausman EM, Jensen-Doss A, Hawley KM. Assessment Practices of Child Clinicians. Assessment. 2017;24(2):210–21. doi:10.1177/1073191115604353 PubMed PMID: 26341574; PubMed Central PMCID: PMC6127860. 26341574 10.1177/1073191115604353 PMC6127860

[3] Jensen-Doss A, Youngstrom EA, Youngstrom JK, Feeny NC, Findling RL. Predictors and moderators of agreement between clinical and research diagnoses for children and adolescents. J Consult Clin Psychol. 2014;82(6):1151–62. 10.1037/a0036657. 24773574 10.1037/a0036657 PMC4278746

[4] Leffler JM, Riebel J, Hughes HM. A Review of Child and Adolescent Diagnostic Interviews for Clinical Practitioners. Assessment. 2015;22(6):690–703. 10.1177/1073191114561253. 25520212 10.1177/1073191114561253

[5] Nilsen TS, Eisemann M, Kvernmo S. Predictors and moderators of outcome in child and adolescent anxiety and depression: a systematic review of psychological treatment studies. Eur Child Adolesc Psychiatry. 2013;22(2):69–87. 10.1007/s00787-012-0316-3. 22923065 10.1007/s00787-012-0316-3

[6] Hansen BH, Oerbeck B, Skirbekk B, Kristensen H. Non-obsessive-compulsive anxiety disorders in child and adolescent mental health services–Are they underdiagnosed, and how accurate is referral information? Nord J Psychiatry. 2016;70(2):133–9. 10.3109/08039488.2015.1061053. 26179992 10.3109/08039488.2015.1061053

[7] Angold A, Costello EJ. Nosology and measurement in child and adolescent psychiatry. J Child Psychol Psychiatry. 2009;50(1–2):9–15. 10.1111/j.1469-7610.2008.01981.x. 19175818 10.1111/j.1469-7610.2008.01981.x

[8] Basco MR, Bostic JQ, Davies D, Rush AJ, Witte B, Hendrickse W, et al. Methods to Improve Diagnostic Accuracy in a Community Mental Health Setting. AJP. 2000;157(10):1599–605. 10.1176/appi.ajp.157.10.1599. 10.1176/appi.ajp.157.10.1599 11007713

[9] Kaufman J, Birmaher B, Brent D, Rao U, Flynn C, Moreci P, et al. Schedule for Affective Disorders and Schizophrenia for School-Age Children-Present and Lifetime Version (K-SADS-PL): initial reliability and validity data. J Am Acad Child Adolesc Psychiatry. 1997;36(7):980–8. 10.1097/00004583-199707000-00021. 9204677 10.1097/00004583-199707000-00021

[10] Cottin M, Blum K, Konjufca J, Quevedo Y, Kaaya S, Behn A, et al. Digital use of standardised assessment tools for children and adolescents: can available paper-based questionnaires be used free of charge in electronic format? BMC Psychiatry. 2022;22(1):379. 10.1186/s12888-022-04023-w. 35659275 10.1186/s12888-022-04023-w PMC9166519

[11] Center for Clinical Research Västmanland. The Electronic Psychiatric Semi-structured Interview for Children and Adolescents (EPSI-C) [Internet]. 2025 [cited 2025 Dec 8]. Available from: https://regionvastmanland.se/vardgivare/forskning-och-utbildning/forskning/salve/

[12] American Psychiatric Association. Diagnostic and statistical manual of mental disorders. 5th ed. Arlington, VA: American Psychiatric Publishing; 2013.

[13] Youngstrom EA, Choukas-Bradley S, Calhoun CD, Jensen-Doss A. Clinical Guide to the Evidence-Based Assessment Approach to Diagnosis and Treatment. Cogn Behav Pract. 2015;22(1):20–35. 10.1016/j.cbpra.2013.12.005.

[14] Polit DF, Beck CT, Owen SV. Is the CVI an acceptable indicator of content validity? Appraisal and recommendations. Res Nurs Health. 2007;30(4):459–67. 10.1002/nur.20199. 17654487 10.1002/nur.20199

[15] Haynes SN, Richard DCS, Kubany ES. Content validity in psychological assessment: A functional approach to concepts and methods. Psychol Assess. 1995;7(3):238–47. 10.1037/1040-3590.7.3.238. Located at: 1996-03400-001.

[16] Tavakol M, Dennick R. Making sense of Cronbach’s alpha. Int J Med Educ. 2011;2:53–5. 10.5116/ijme.4dfb. 8dfd PubMed PMID: 28029643; PubMed Central PMCID: PMC4205511. 28029643 10.5116/ijme.4dfb.8dfd PMC4205511

[17] Cronbach LJ. Coefficient alpha and the internal structure of tests. Psychometrika. 1951;16(3):297–334. 10.1007/BF02310555.

[18] Streiner DL. Starting at the Beginning: An Introduction to Coefficient Alpha and Internal Consistency. J Pers Assess. 2003;80(1):99–103. doi:10.1207/S15327752JPA8001_18 PubMed PMID: 12584072. 12584072 10.1207/S15327752JPA8001_18

[19] Center for Clinical Research Västmanland, Uppsala University. The Electronic Psychiatric Semi-structured Interview for Children and Adolescents (EPSI-C) [Internet]. 2026 [cited 2026 Jan 15]. Available from: https://www.uu.se/en/centre/clinical-research-vastmanland/research/salve-english

[20] Sheehan DV, Sheehan KH, Shytle RD, Janavs J, Bannon Y, Rogers JE, et al. Reliability and validity of the Mini International Neuropsychiatric Interview for Children and Adolescents (MINI-KID). J Clin Psychiatry. 2010;71(3):313–26. 10.4088/JCP.09m05305whi. 20331933 10.4088/JCP.09m05305whi

[21] Polit DF, Beck CT. The content validity index: Are you sure you know what’s being reported? critique and recommendations. Res Nurs Health. 2006;29(5):489–97. 10.1002/nur.20147. 16977646 10.1002/nur.20147

[22] Almanasreh E, Moles R, Chen TF. Evaluation of methods used for estimating content validity. Res Social Adm Pharm. 2019;15(2):214–21. 10.1016/j.sapharm.2018.03. .066 PubMed PMID: 29606610. 29606610 10.1016/j.sapharm.2018.03.066

[23] Nunnally JC, Bernstein IH. Psychometric theory. 3rd ed. New York: McGraw-Hill; 1994.

[24] Samuels P. Statistical Methods – Scale reliability analysis with small samples [Internet]. Birmingham City University, Centre for Academic Success; 2017. Available from: https://www.open-access.bcu.ac.uk/6077/1/__staff_shares_storage%20500mb_Library_ID112668_Stats%20Advisory_New%20Statistics%20Workshops_17ReliabilityAnalysis_ReliabilitySmallSamples3.pdf

[25] Kariuki SM, Newton CRJC, Abubakar A, Bitta MA, Odhiambo R, Phillips Owen J. Evaluation of Psychometric Properties and Factorial Structure of ADHD Module of K-SADS-PL in Children From Rural Kenya. J Atten Disord. 2020;24(14):2064–71. 10.1177/1087054717753064. 29392964 10.1177/1087054717753064 PMC7549293

[26] Giannopoulos E, Hilsenroth M. Psychometric characteristics of DSM-5 eating disorder diagnostic criteria: support for a transdiagnostic approach. J Eat Disord. 2026;14(1):24. 10.1186/s40337-025-01512-7. 41545883 10.1186/s40337-025-01512-7 PMC12829015

[27] Malkewitz CP, Schwall P, Meesters C, Hardt J. Estimating reliability: A comparison of Cronbach’s α, McDonald’s ωt and the greatest lower bound. Social Sci Humanit Open. 2023;7(1):100368. 10.1016/j.ssaho.2022.100368.

