# Supplementary material for: The Electronic Psychiatric Semi-structured Interview for Children and Adolescents (EPSI-C): development and psychometric evaluation of an open-access DSM-5-based diagnostic instrument
Source: Child Adolesc Psychiatry Ment Health. 2026 May 6;20:67. doi: 10.1186/s13034-026-01094-5 (PMC13151297; doi:10.1186/s13034-026-01094-5)
Supplement: Supplementary file 1 — Supplementary Material 1 [file 13034_2026_1094_MOESM1_ESM.docx]

Supplementary Material for the manuscript:

**The Electronic Psychiatric Semi-structured Interview for Children and Adolescents (EPSI-C): Development and Psychometric Evaluation of an Open-Access DSM-5-based Diagnostic Instrument**

**Journal: Child and Adolescent Psychiatry and Mental Health**

Authors: Susanne Olofsdotter ^a,b^, Melpomeni Dragou ^a,b^, Johan Isaksson ^b,c^, Kent W Nilsson ^a,b,d^, Sofia Vadlin ^a^, Maria Hedqvist ^a,e^

^a^ Center for Clinical Research Västmanland, Uppsala University, Västerås, Sweden

^b^ Child and Adolescent Psychiatry, Department of Medical Sciences, Uppsala University, Sweden

^c^ Center of Neurodevelopmental Disorders (KIND), Centre for Psychiatry Research; Department of Women's and Children's Health, Karolinska Institutet & Stockholm Health Care Services, Region Stockholm, Stockholm, Sweden

^d^ School of Health, Care and Social Welfare, Division of Public Health Sciences, Mälardalen University, Västerås, Sweden

^e^ Department of Psychology, Uppsala University, Sweden

Correspondence: [**susanne.olofsdotter@regionvastmanland.se**](mailto:susanne.olofsdotter@regionvastmanland.se) Center for Clinical Research, Västmanland County Hospital Västerås, 721 89 Västerås, Sweden

**MODULE: GENERALIZED ANXIETY DISORDER**

Information for the interviewer:

GENERALIZED ANXIETY DISORDER is characterised by excessive or disproportionate worry and anxiety about several different events or activities. The anxiety may be anticipatory and linked to everyday situations that could happen in the future, e.g., concerns or “what if”-thoughts about family/health, school, the future, finances. It can also be about how different events have unfolded or how the child has acted in a particular situation. The anxiety is difficult to control and can lead to muscle tension, restlessness, hyperactivity of the sympathetic autonomic nervous system (e.g., palpitations), nervousness, difficulty concentrating, irritability, or difficulty sleeping.

For diagnosis, the worry should be disabling and persistent, occurring more days than not for at least 6 months.

**SCREEN QUESTION:**

1. **Assess whether the child experiences excessive or disproportionate anxiety and worry about multiple events or activities, occurring more days than not for at least 6 months.**

**
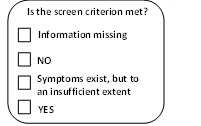
**

Probing questions:

- *Do you often have worrying thoughts? Do you feel a constant sense of worry or anxiety? Does it make you feel very stressed?*
- *Do you worry about many different things? For example: what might happen to you, your parents’ health, school, the future, money, the environment or the world, or that something bad might happen?*
- *Do you often have anxious thoughts that start with “What if...?”. Do you sometimes feel like you are overthinking everything?*
- *What kinds of things do you usually worry about?*
- *How often do you worry about these things? Is it almost every day? How many times in a day do you worry about these things?*
- *Do you feel that anxiety makes it hard for you to focus on what you are doing?*
- *Do other people say that you worry too much?*
- *How long have you been feeling this way? Has it been going on for several months? Has it lasted for six months or more?*

**MODULE: GENERALIZED ANXIETY DISORDER**

1. *
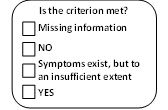
* **Assess whether the child has difficulty controlling the worry.**

Probing questions:

- *Do you find it hard to stop worrying?*
- *Can you stop worrying when you need to focus? For example, during schoolwork, homework, or when you are with other people.*
- *Do you do anything to try to control your worry? For example, asking others for reassurance about your worries, searching online, or distracting yourself?*

1. *
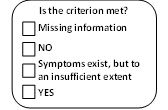
* **Assess whether the child’s worry is associated with any of the following symptoms (at least one of which must have been present for more days than not during the past 6 months). Tick all that apply:**

- Restlessness, feeling keyed up or on edge
- Being easily fatigued
- Difficulty concentrating, mind goes blank
- Irritability
- Muscle tension
- Sleep disturbance

1. *
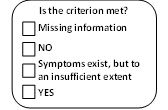
* **Assess whether the child’s anxiety, worry, or physical symptoms cause clinically significant distress or impairment (e.g., socially, at school, within the family, or in other areas).**

Probing questions*:*

- *How does this affect your life? Does it cause problems? In what way?*
- *Does it cause problems in your free time? When you spend time with friends?*
- *Is your schoolwork affected? How?*
- *Does this mean that you need a lot of support or help from your family, parents or friends? In what way?*
- *Is your life affected in any other way that I have not asked about?*

1. *
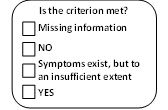
* **Assess whether a substance, such as illegal drugs or medications, or another medical condition can be excluded as the cause of the symptoms.**

Probing questions:

- *Do you use or have you used alcohol or other drugs that could cause your symptoms?*
- *Have you been (physically) ill while having these symptoms?*
- *Have you taken any medication that could be causing your symptoms?*
