# Supplementary material for: The Electronic Psychiatric Semi-structured Interview for Children and Adolescents (EPSI-C): development and psychometric evaluation of an open-access DSM-5-based diagnostic instrument
Source: Child Adolesc Psychiatry Ment Health. 2026 May 6;20:67. doi: 10.1186/s13034-026-01094-5 (PMC13151297; doi:10.1186/s13034-026-01094-5)
Supplement: Supplementary file 2 — Supplementary Material 2 [file 13034_2026_1094_MOESM2_ESM.docx]

Supplementary Material for the manuscript:

**The Electronic Psychiatric Semi-structured Interview for Children and Adolescents (EPSI-C): Development and Psychometric Evaluation of an Open-Access DSM-5-based Diagnostic Instrument**

**Journal: Child and Adolescent Psychiatry and Mental Health**

Authors: Susanne Olofsdotter ^a,b^, Melpomeni Dragou ^a,b^, Johan Isaksson ^b,c^, Kent W Nilsson ^a,b,d^, Sofia Vadlin ^a^, Maria Hedqvist ^a,e^

^a^ Center for Clinical Research Västmanland, Uppsala University, Västerås, Sweden

^b^ Child and Adolescent Psychiatry, Department of Medical Sciences, Uppsala University, Sweden

^c^ Center of Neurodevelopmental Disorders (KIND), Centre for Psychiatry Research; Department of Women's and Children's Health, Karolinska Institutet & Stockholm Health Care Services, Region Stockholm, Stockholm, Sweden

^d^ School of Health, Care and Social Welfare, Division of Public Health Sciences, Mälardalen University, Västerås, Sweden

^e^ Department of Psychology, Uppsala University, Sweden

Correspondence: [**susanne.olofsdotter@regionvastmanland.se**](mailto:susanne.olofsdotter@regionvastmanland.se) Center for Clinical Research, Västmanland County Hospital Västerås, 721 89 Västerås, Sweden

**Total clinical sample (N = 3,506)**

Unique, consecutive patients (5 - 18 years) assessed during face-to-face routine assessments within a child and adolescent psychiatric outpatient clinic in Västmanland County, Sweden. All patients were systematically screened with a core set of EPSI-C modules, including those for anxiety disorders, depression, posttraumatic stress disorder, obsessive-compulsive disorder, psychosis, ADHD, autism, and suicidality, irrespective of referral reason. Other modules were assessed only when clinically indicated. Full diagnostic modules were administered conditionally, triggered upon a positive screening result.

Figure S1. Inclusion flowchart for internal consistency analyses. Modules in bold text were included in analyses. Modules marked with * were part of a core set of modules, assessed for all patients. Other modules were assessed only when clinically indicated.

**EPSI-C modules: Behavioral, substance use, and eating disorders**

- **ANOREXIA NERVOSA: PS: n = 44; Excl n = 3; FS n = 41**
- BULIMIA NERVOSA: PS: n = 7; Excl n = 1; FS n = 6
- BINGE-EATING DISORDER: PS: n = 4 ; Excl n = 1; FS n = 3
- **OPPOSITIONAL DEFIANT DISORDER: PS: n =83 ; Excl n = 9; FS n = 74**
- CONDUCT DISORDER: PS: n = 16; Excl n = 0; FS n = 16
- SCREEN FOR SUBSTANCE USE DISORDERS: PS: n = 9; Excl n = 5; FS n = 4

**EPSI-C modules: Neurodevelopmental disorders**

- **ADHD^*^: PS: n = 2,533 ; Excl n = 181; FS n = 2,352**
- **AUTISM SPECTRUM DISORDER^*^: PS: n = 1,489 ; Excl n = 81; FS n = 1,408**
- **TOURETTE’S DISORDER/TICS: PS: n = 60 ; Excl n = 4; FS n = 56**

**EPSI-C modules: Anxiety disorders**

- **SPECIFIC PHOBIA: PS: n = 67 ; Excl n = 3; FS n = 64**
- **SEPARATION ANXIETY DISORDER^*^: PS: n = 464 ; Excl n = 34; FS n = 430**
- **SOCIAL ANXIETY DISORDER^*^: PS: n = 1,041 ; Excl n = 44; FS n = 997**
- **PANIC DISORDER^*^: PS: n = 505 ; Excl n = 10; FS n = 495**
- **AGORAPHOBIA^*^: PS: n = 151; Excl n = 4; FS n = 147**
- **GENERALIZED ANXIETY DISORDER^*^: PS: n = 800; Excl n = 13; FS n = 787**

**EPSI-C modules: Mood, trauma, suicidality disorders**

- **OBSESSIVE–COMPULSIVE DISORDER^*^: PS: n = 214; Excl n = 16; FS n = 198**
- **PTSD^*^: PS: n = 166 ; Excl n = 72; FS n = 94**
- **MAJOR DEPRESSIVE EPISODE^*^: PS: n = 1,071 ; Excl n = 38; FS n = 1,033**
- PERSISTENT DEPRESSION: PS: n = 14; Excl n = 1; FS n = 13
- MANIC/HYPOMANIC EPISODE: PS: n = 20; Excl n = 0; FS n = 20
- **SCREEN FOR PSYCHOTIC DISORDERS^*^: PS: n = 70 ; Excl n = 9; FS n = 61**
- **SCREEN FOR SUICIDALITY^*^ (total sample screened): Excl n = 152; FS n = 3,354**

**EPSI-C module inclusion in internal consistency analyses**

Module inclusion criteria: at least 30 patients with complete data for all items.

Positive screen **(PS) =** full module assessed; Excluded **(Excl)=** number of patients excluded due to missing data

Final sample **(FS)=** number of patients included in internal consistency analysis

Number of patients with negative screen/no clinical indication (full module skipped) = **3,506 – PS**
